# Supplementary material for: Spatiotemporal proteomic atlas of multiple brain regions across early fetal to neonatal stages in cynomolgus monkey
Source: Nat Commun. 2023 Jul 3;14:3917. doi: 10.1038/s41467-023-39411-7 (PMC10317979; doi:10.1038/s41467-023-39411-7)
Supplement: Supplementary file 1 — Supplementary Information [file 41467_2023_39411_MOESM1_ESM.pdf]

Supplemental materials for

Spatiotemporal proteomic atlas of multiple brain regions across early fetal to neonatal stages in cynomolgus monkey

Jingkuan Wei[1,2,#], Shaoxing Dai[1,2,#], Yaping Yan[1,2,#], Shulin Li[1], Pengpeng Yang[1], Ran Zhu[1], Tianzhuang Huang[1,2], Xi Li[1,2], Yanchao Duan[1,2], Zhengbo Wang[1,2,*], Weizhi Ji[1,2,3,*], Wei Si[1,2,3,*]

[1]State Key Laboratory of Primate Biomedical Research; Institute of Primate Translational Medicine, Kunming University of Science and Technology, Kunming, Yunnan, 650500, China

[2]Yunnan Key Laboratory of Primate Biomedical Research, Kunming, Yunnan, 650500, China

[3]Chinese Primate Biomedical Research Alliance (CPBRA)

[#] These authors contributed equally to this work.

*Correspondence: wangzb@lpbr.cn (Z. Wang), wji@lpbr.cn (W. Ji), siw@lpbr.cn (W. Si)

The supplemental materials include:

Supplementary Figure 1. Determination of the embryonic days and detection of embryonic development by ultrasound. Determination of the embryonic days and detection of embryonic development by ultrasound.

Supplementary Figure 2. Enrichment analysis of the stage-specific marker proteins in Figure 2E.

Supplementary Figure 3. Comparison of biological processes enriched by CB marker proteins at the four stages (Related to Figure 3C).

Supplementary Figure 4. The dynamic changes of proteins associated with medulloblastomas in cerebellum at the four developmental stages.

Supplementary Figure 5. Protein expression and KEGG pathway enrichment of Mfuzz clusters

Supplementary Figure 6. Heatmap showing Pearson correlations among the five brain regions for each protein in the top 6-10 abundant protein families under the three pairwise comparisons.

Supplementary Figure 7. Different changes of RNA and protein levels between prenatal and postnatal.

Supplementary Figure 8. Biological processes enriched by the 1-3 types genes in cortical (A) and subcortical regions (B).

Supplementary Figure 9. Biological processes enriched by the 1-3 types genes in cerebellum.

Supplementary Figure 10. Biological processes enriched by the 4-6 types genes in cortical (A) and subcortical regions (B)

Supplementary Figure 11. Biological processes enriched by the 4-6 types genes in cerebellum.

Supplementary Table 1. Information for the 156 samples in this study.

Supplementary Figures

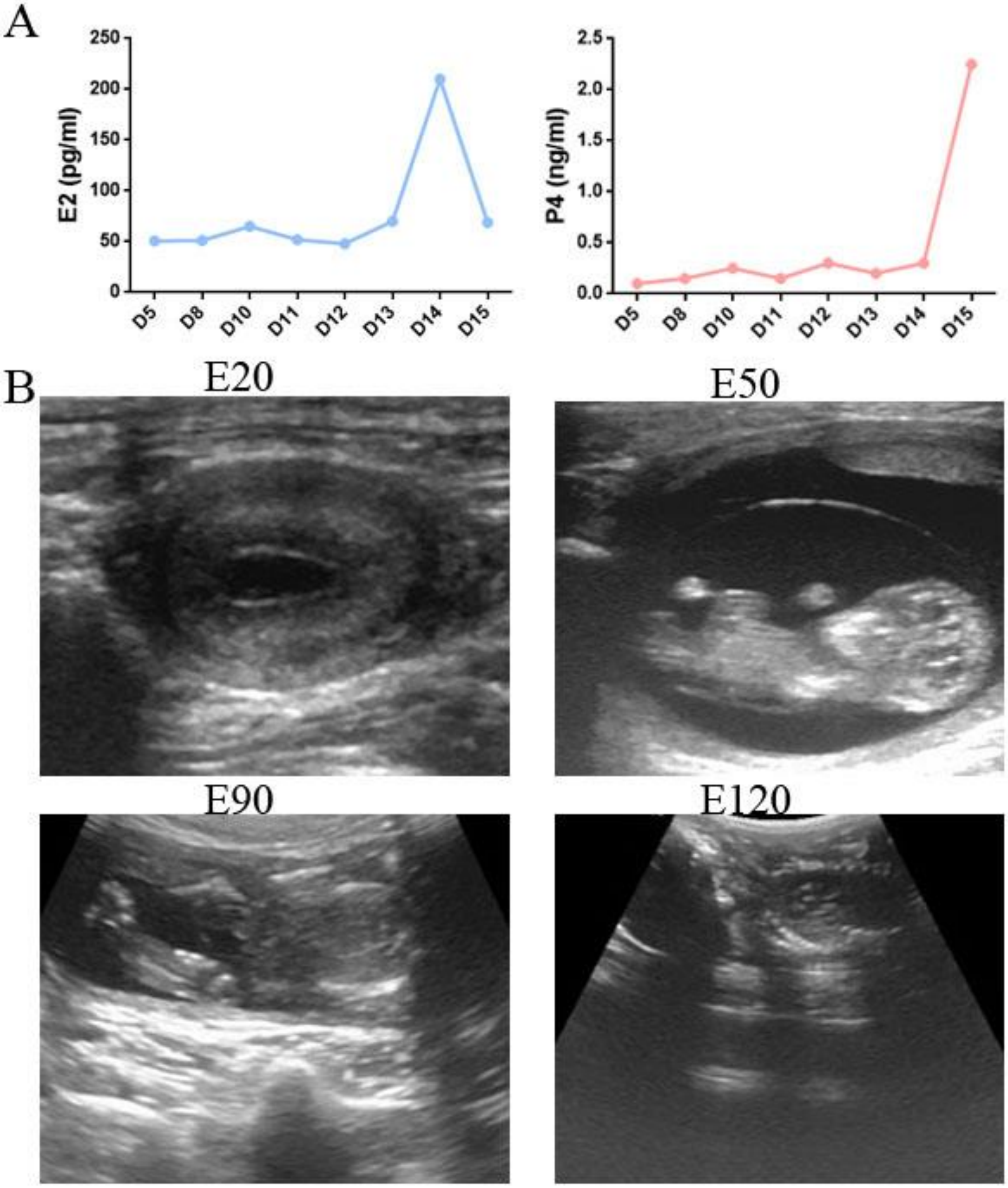

Supplementary Figure 1. Determination of the embryonic days and detection of embryonic development by ultrasound. (**A**) The ovulation time and conception time of female monkeys were determined by detecting the concentrations of estradiol (E2) and progesterone (P4) in peripheral blood. (**B**) Detection of embryonic development by ultrasound, including ultrasound images of 20 days post-fertilization (F20), 50 days post-fertilization (F50), 90 days post-fertilization (F90) and 120 days post-fertilization (F120).

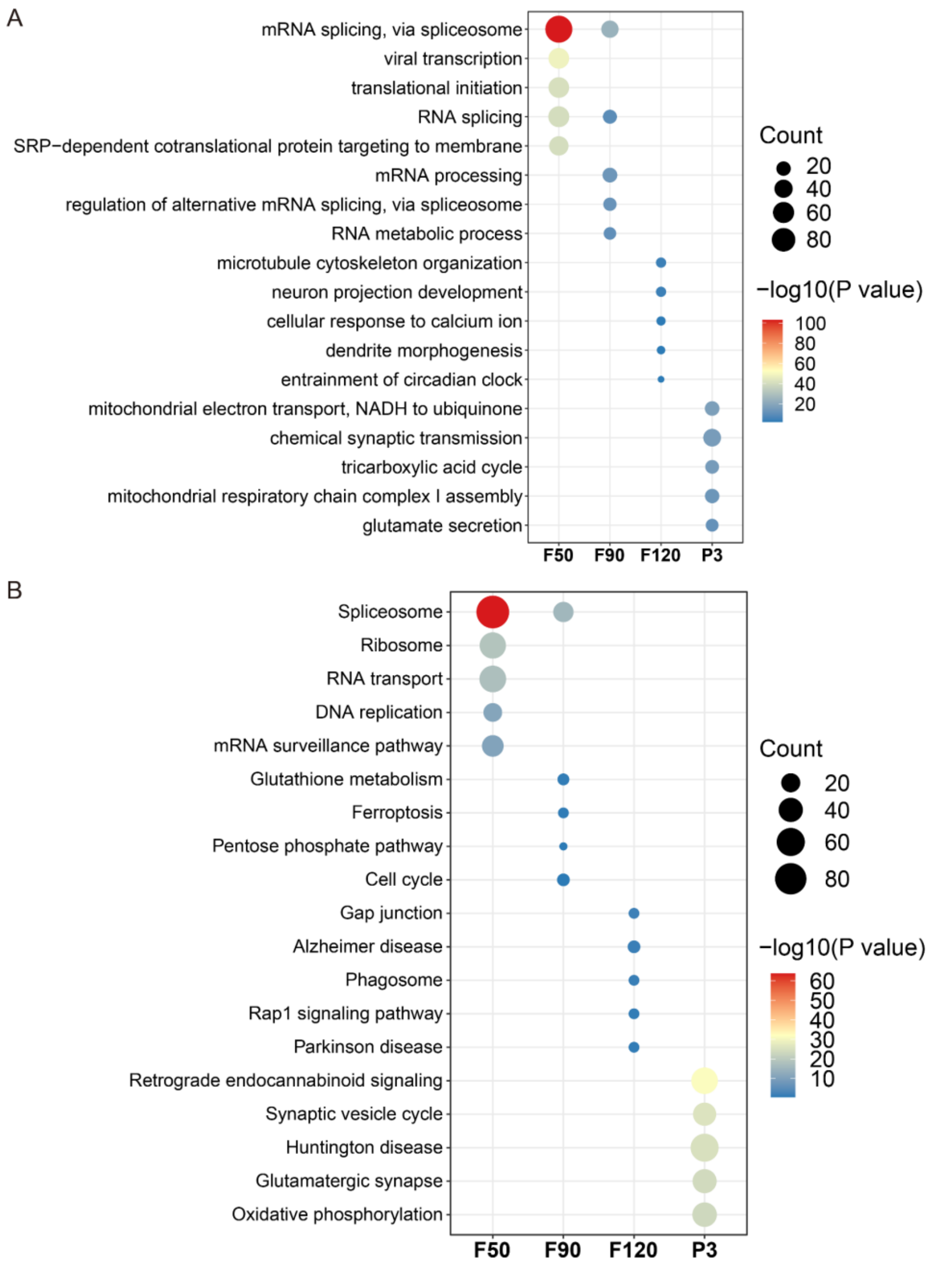

Supplementary Figure 2. Enrichment analysis of the stage marker proteins in Figure 2E. (**A**) Dotplot showing the biological processes enriched by the stage marker proteins in the indicated stages. (**B**) Dotplot showing the KEGG pathways enriched by the stage marker proteins in the indicated stages. The color shade and dot size indicate the P value and the number of genes, respectively. The P values were calculated by two-sided Fisher's exact test. Source data are provided as a Source Data file.

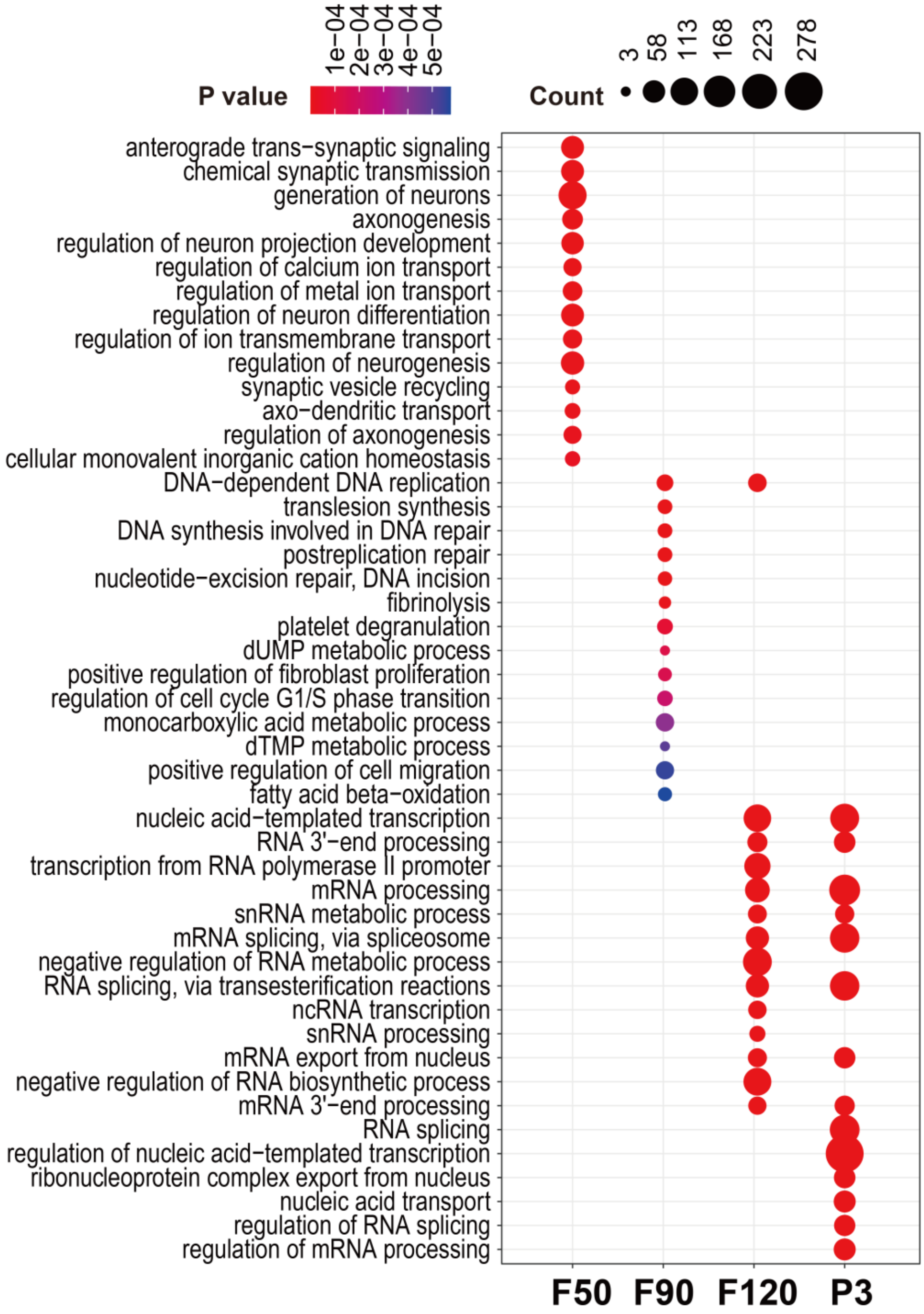

Supplementary Figure 3. Comparison of biological processes enriched by CB marker proteins at the four stages (Related to Figure 3C). The color shade and dot size indicate the P value and the number of genes, respectively. The P values were calculated by two-sided Fisher's exact test. Source data are provided as a Source Data file.

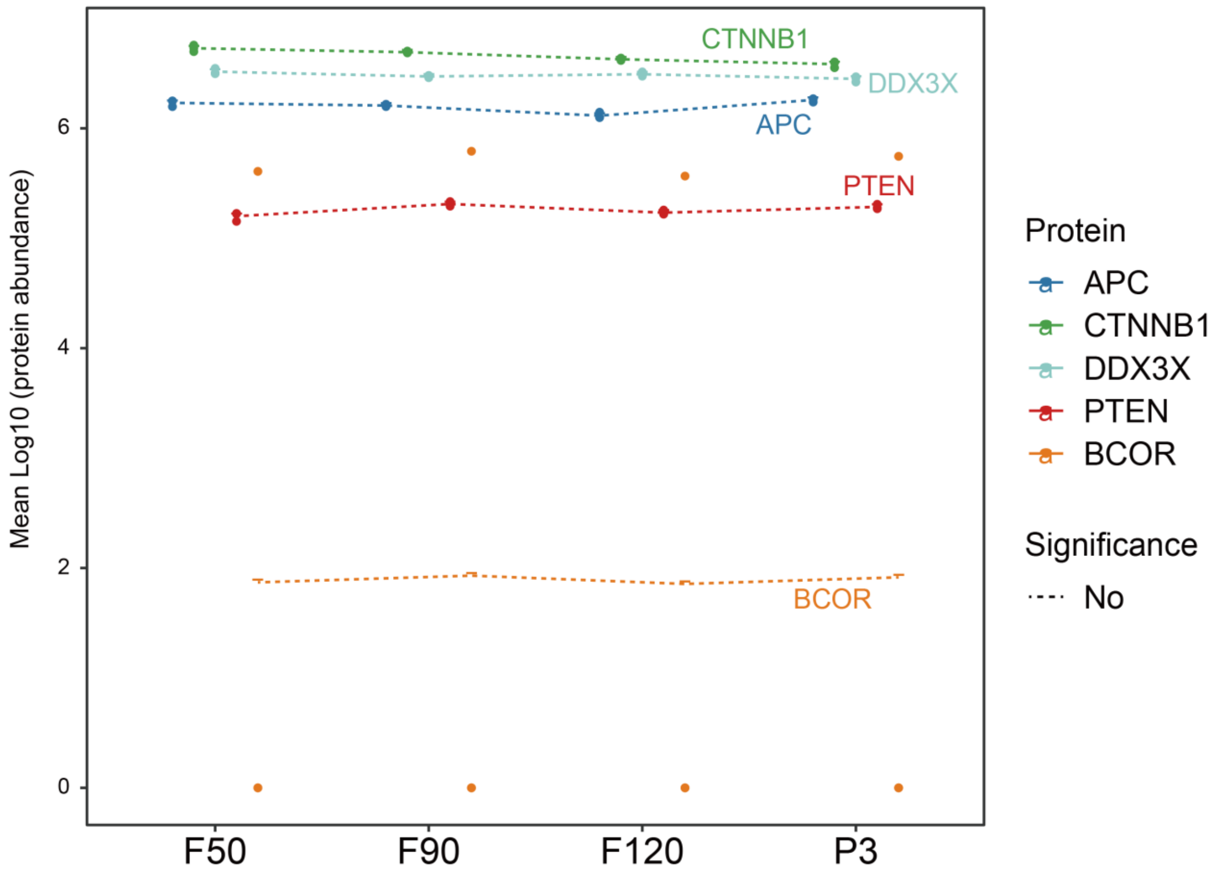

Supplementary Figure 4. The dynamic changes of proteins associated with medulloblastomas in cerebellum at the four developmental stages. This figure is related to Figure 3.). The line chart showing the abundance change of proteins (APC, CTNNB1, DDX3X, PTEN, BCOR). The proteins were indicated by different colors. The dashed line indicates no significant difference between any two adjacent stages. The solid line indicates that there is at least one significant difference between two adjacent stages. The P values were calculated by two-sided student's t-test test. Source data are provided as a Source Data file.

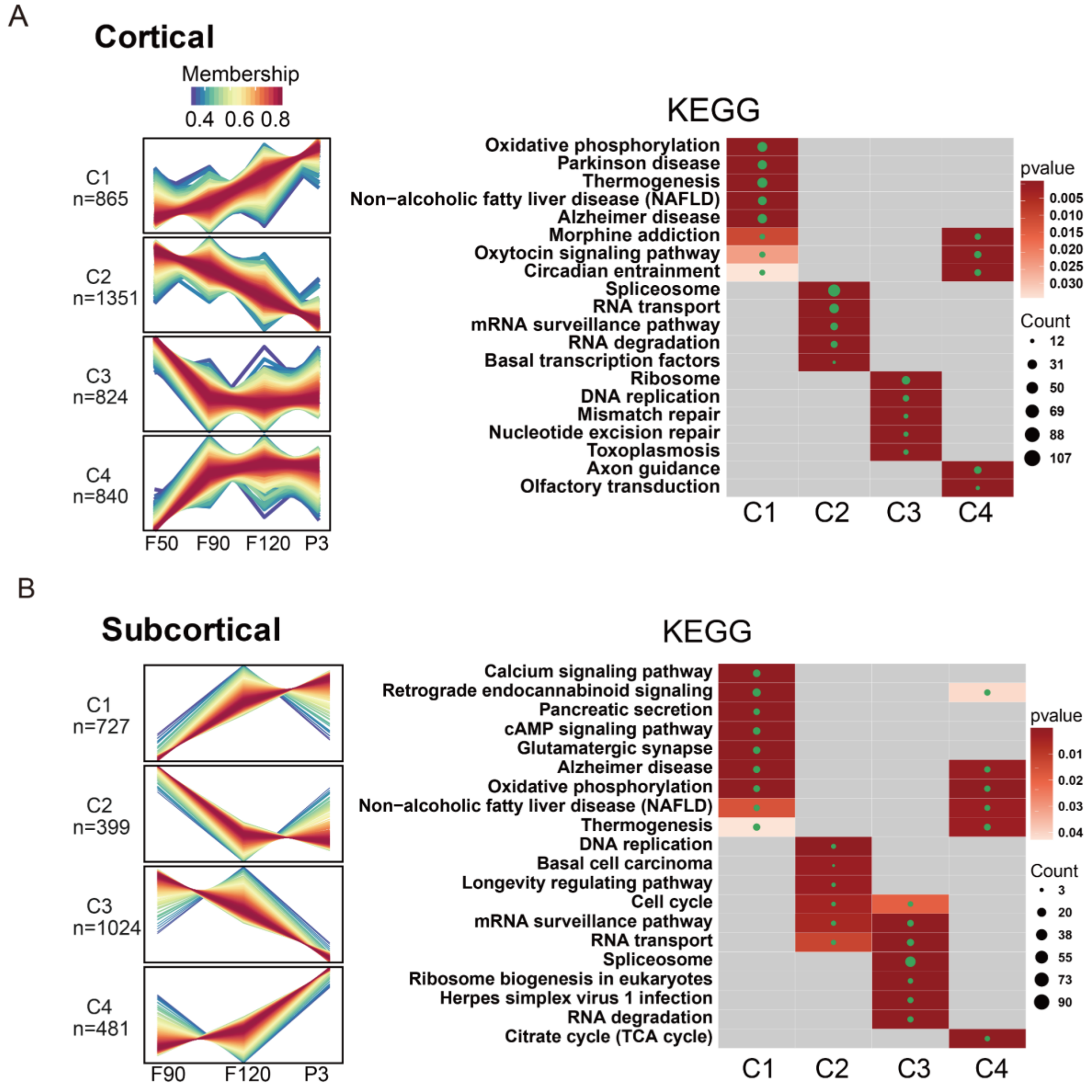

Supplementary Figure 5. Protein expression and KEGG pathway enrichment of Mfuzz clusters. Mfuzz analysis determined four clusters of proteins across the four stages in cortical and subcortical regions, respectively. The protein expression pattern of four clusters across the four stages in cortical (**A**) and subcortical region (**B**) were shown. The KEGG pathways enriched by proteins in the four clusters were shown by Dotplot. The membership with color varying from blue to red represents the suitable degree of proteins with the change trend of the cluster. The P values were calculated by two-sided Fisher's exact test (**A**, **B**). Source data are provided as a Source Data file.

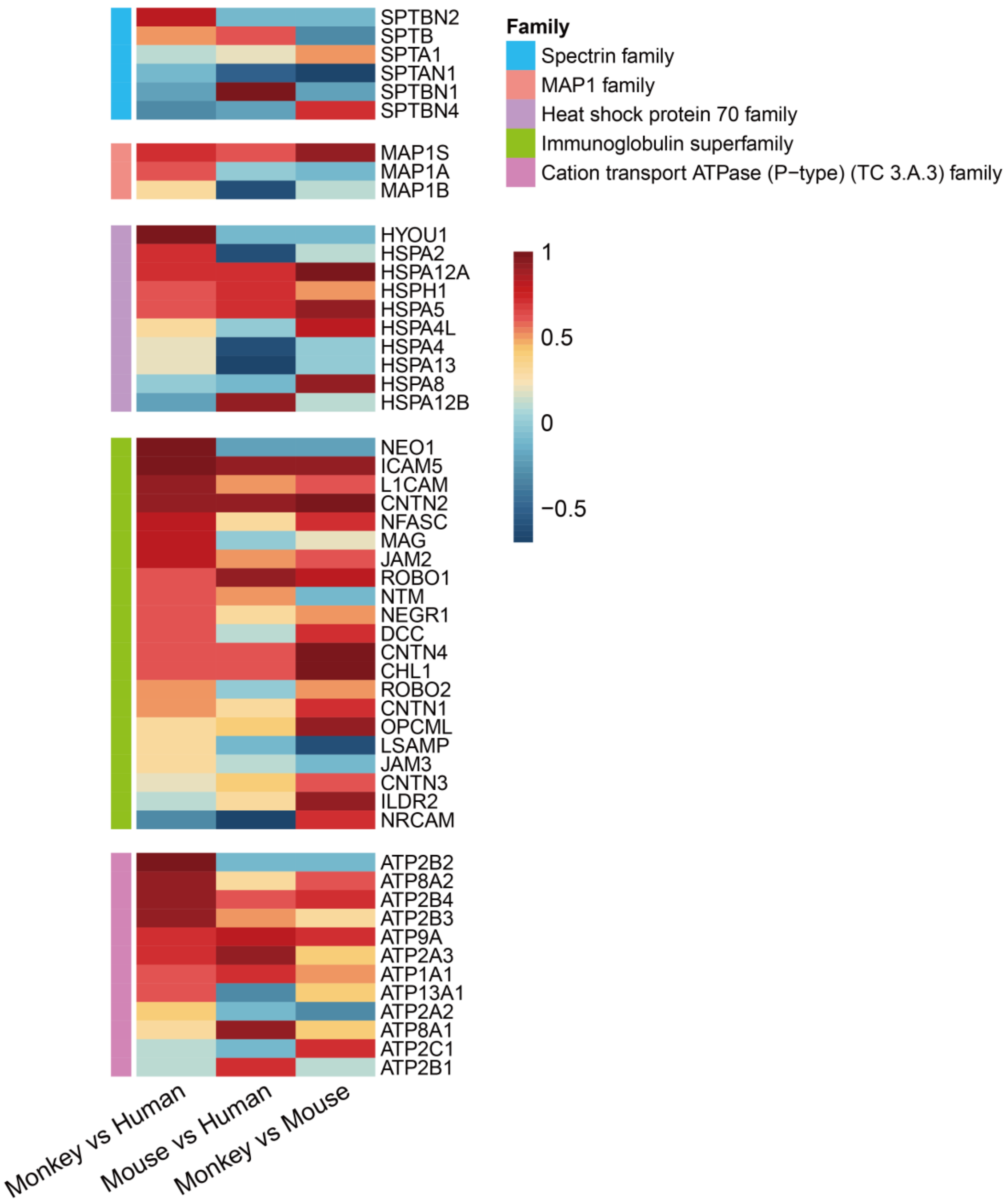

Supplementary Figure 6. Heatmap showing Pearson correlations among the five brain regions for each protein in the top 6-10 abundant protein families under the three pairwise comparisons. The color shade indicates the Pearson correlations. Source data are provided as a Source Data file.

A

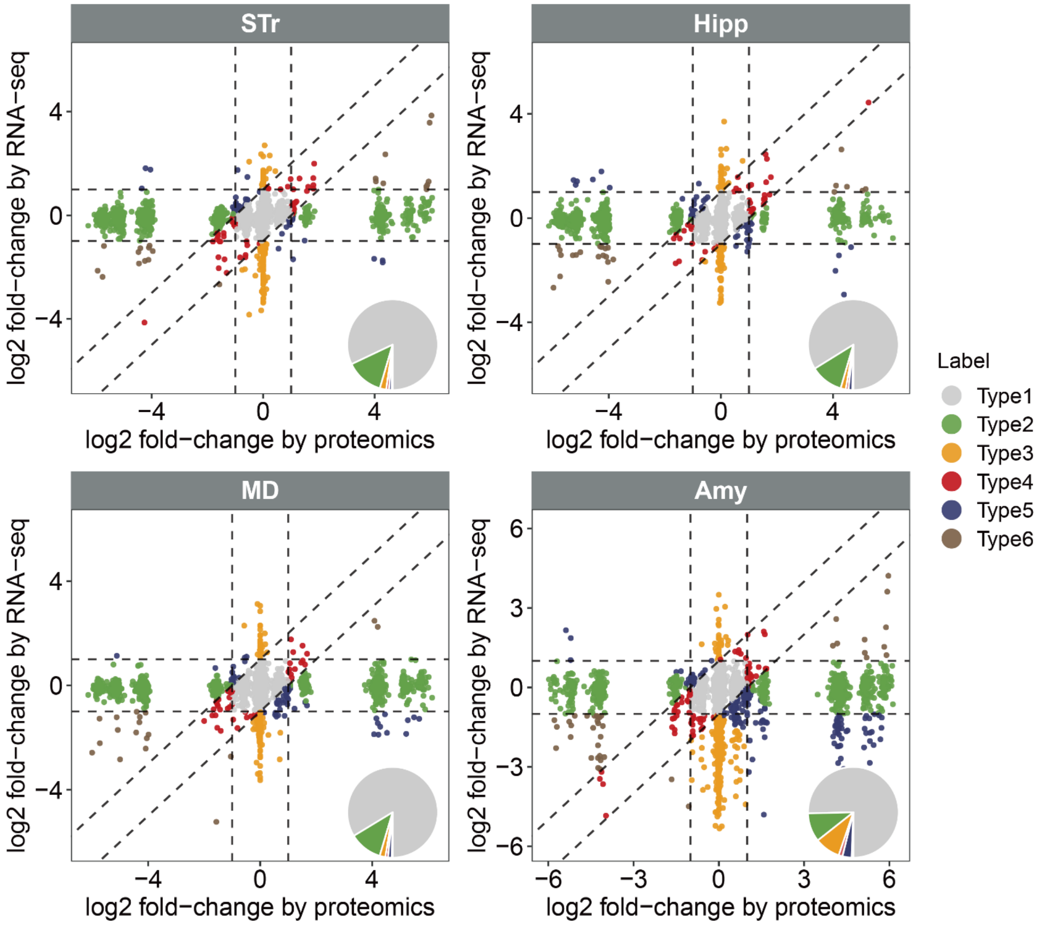
STr
Hipp
MD
Amy
log2 fold−change by RNA−seq
log2 fold−change by proteomics
Label
Type1
Type2
Type3
Type4
Type5
Type6

B

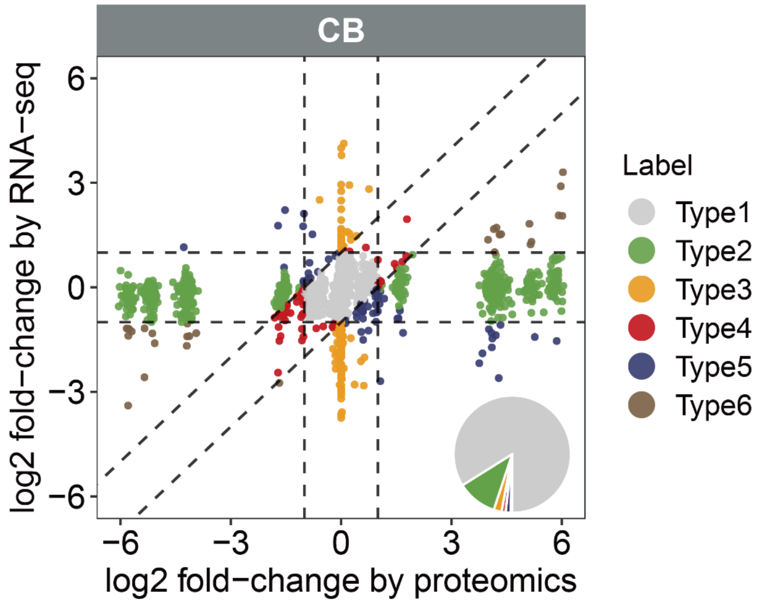
CB
log2 fold−change by RNA−seq
log2 fold−change by proteomics
Label
Type1
Type2
Type3
Type4
Type5
Type6

Supplementary Figure 7. Different changes of RNA and protein levels between prenatal and postnatal. The scatter plot showing the fold changes (FC) between prenatal and postnatal for the RNA-seq and proteomics in subcortical regions (**A**) and cerebellum (**B**). Based on FC of RNA and protein levels, all genes can be divided into 6 types as defined in the section of Methods. The 6 types genes were indicated by different colors. Inset pie charts illustrate the relative percentages of the 6 types genes. CB, cerebellum; STr, striatum; HIpp, hippocampus: MD, medial dorsal nucleus of thalamus; Amy, amygdala. Source data are provided as a Source Data file.

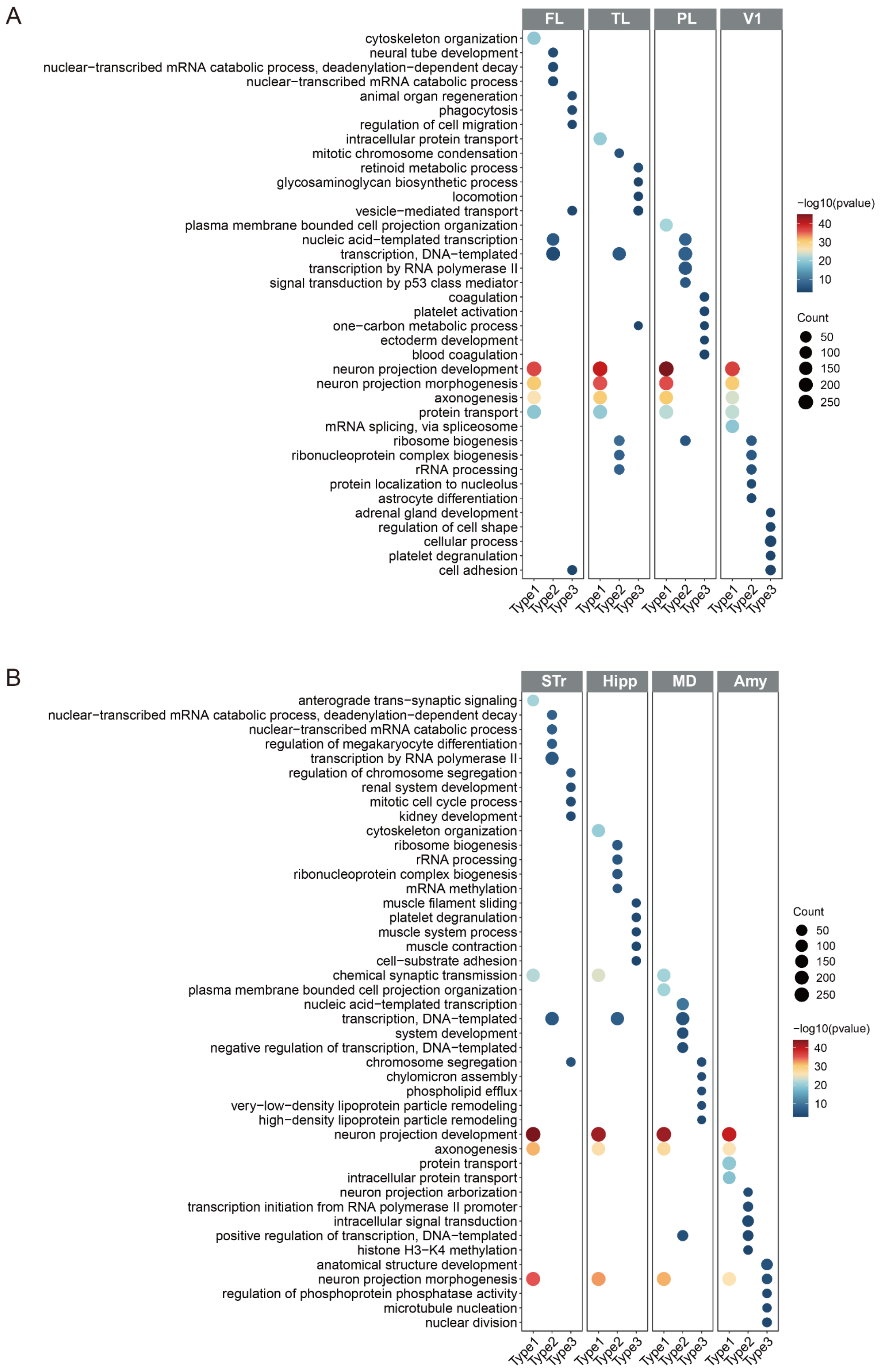
A
FL
TL
PL
V1
cytoskeleton organization
neural tube development
nuclear−transcribed mRNA catabolic process, deadenylation−dependent decay
nuclear−transcribed mRNA catabolic process
animal organ regeneration
phagocytosis
regulation of cell migration
intracellular protein transport
mitotic chromosome condensation
retinoid metabolic process
glycosaminoglycan biosynthetic process
locomotion
vesicle−mediated transport
plasma membrane bounded cell projection organization
nucleic acid−templated transcription
transcription, DNA−templated
transcription by RNA polymerase II
signal transduction by p53 class mediator
coagulation
platelet activation
one−carbon metabolic process
ectoderm development
blood coagulation
neuron projection development
neuron projection morphogenesis
axonogenesis
protein transport
mRNA splicing, via spliceosome
ribosome biogenesis
ribonucleoprotein complex biogenesis
rRNA processing
protein localization to nucleolus
astrocyte differentiation
adrenal gland development
regulation of cell shape
cellular process
platelet degranulation
cell adhesion
Type1
Type2
Type3
−log10(pvalue)
40
30
20
10
Count
50
100
150
200
250
B
STr
Hipp
MD
Amy
anterograde trans−synaptic signaling
nuclear−transcribed mRNA catabolic process, deadenylation−dependent decay
nuclear−transcribed mRNA catabolic process
regulation of megakaryocyte differentiation
transcription by RNA polymerase II
regulation of chromosome segregation
renal system development
mitotic cell cycle process
kidney development
cytoskeleton organization
ribosome biogenesis
rRNA processing
ribonucleoprotein complex biogenesis
mRNA methylation
muscle filament sliding
platelet degranulation
muscle system process
muscle contraction
cell−substrate adhesion
chemical synaptic transmission
plasma membrane bounded cell projection organization
nucleic acid−templated transcription
transcription, DNA−templated
system development
negative regulation of transcription, DNA−templated
chromosome segregation
chylomicron assembly
phospholipid efflux
very−low−density lipoprotein particle remodeling
high−density lipoprotein particle remodeling
neuron projection development
axonogenesis
protein transport
intracellular protein transport
neuron projection arborization
transcription initiation from RNA polymerase II promoter
intracellular signal transduction
positive regulation of transcription, DNA−templated
histone H3−K4 methylation
anatomical structure development
neuron projection morphogenesis
regulation of phosphoprotein phosphatase activity
microtubule nucleation
nuclear division
Type1
Type2
Type3
Count
50
100
150
200
250
−log10(pvalue)
40
30
20
10

Supplementary Figure 8. Biological processes enriched by the 1-3 types genes in cortical (A) and subcortical regions (B). The color shade and dot size indicate the P value and the number of genes, respectively. The P values were calculated by two-sided Fisher's exact test (**A**, **B**). Source data are provided as a Source Data file.

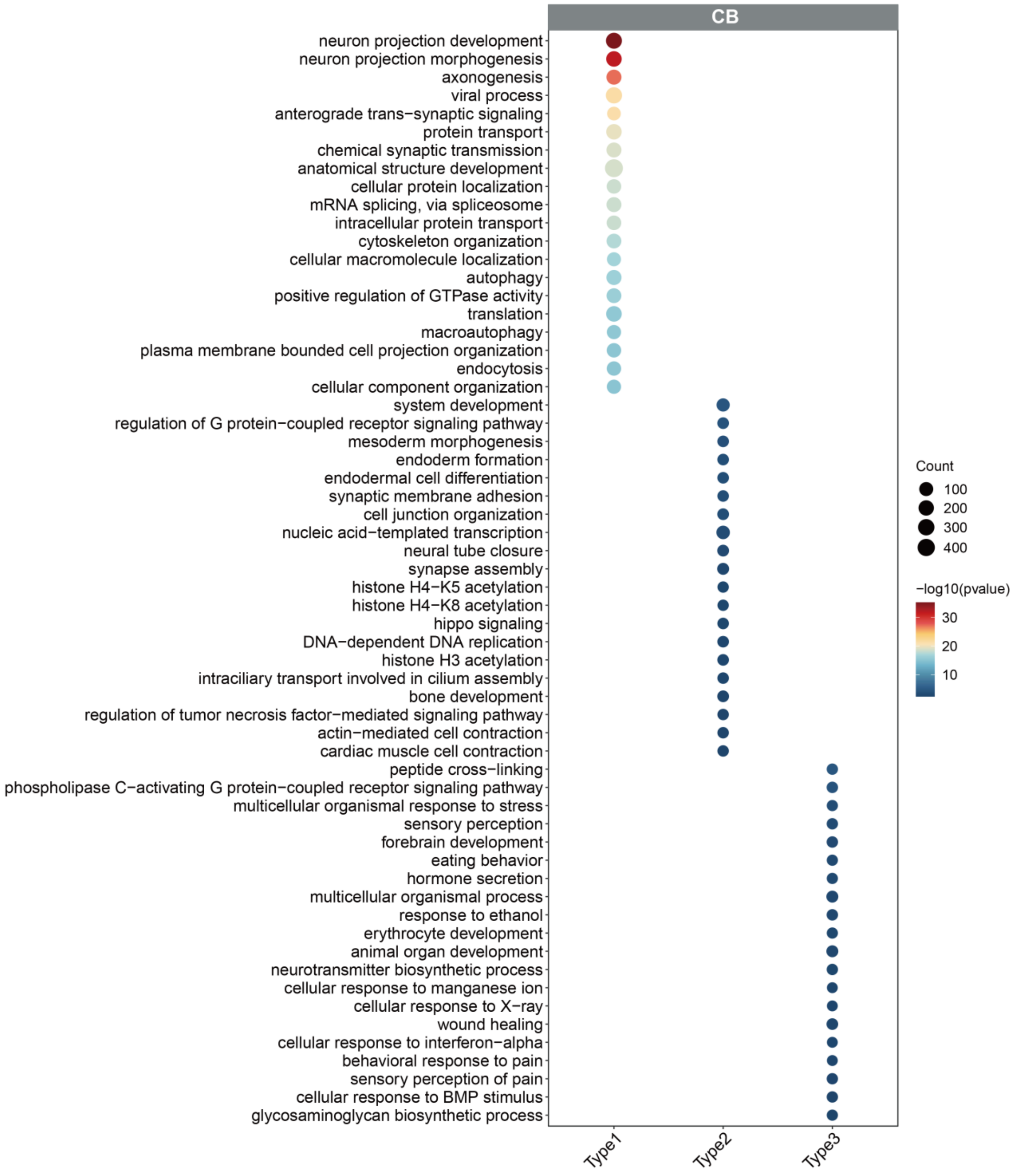

Supplementary Figure 9. Biological processes enriched by the 1-3 types genes in cerebellum. The color shade and dot size indicate the P value and the number of genes, respectively. The P values were calculated by two-sided Fisher's exact test. Source data are provided as a Source Data file.

A

B

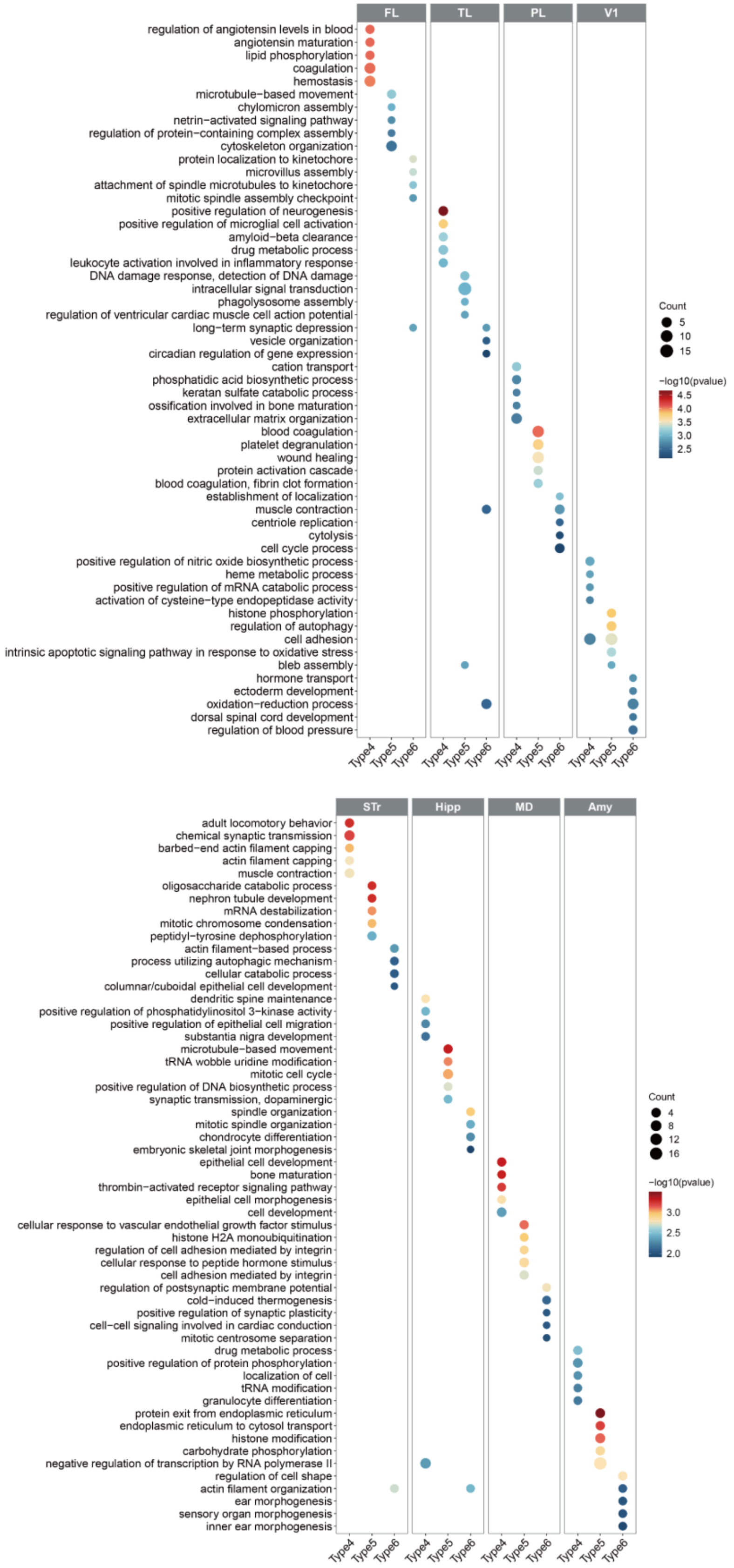
FL
TL
PL
V1
regulation of angiotensin levels in blood
angiotensin maturation
lipid phosphorylation
coagulation
hemostasis
microtubule−based movement
chylomicron assembly
netrin−activated signaling pathway
regulation of protein−containing complex assembly
cytoskeleton organization
protein localization to kinetochore
microvillus assembly
attachment of spindle microtubules to kinetochore
mitotic spindle assembly checkpoint
positive regulation of neurogenesis
positive regulation of microglial cell activation
amyloid−beta clearance
drug metabolic process
leukocyte activation involved in inflammatory response
DNA damage response, detection of DNA damage
intracellular signal transduction
phagolysosome assembly
regulation of ventricular cardiac muscle cell action potential
long−term synaptic depression
vesicle organization
circadian regulation of gene expression
cation transport
phosphatidic acid biosynthetic process
keratan sulfate catabolic process
ossification involved in bone maturation
extracellular matrix organization
blood coagulation
platelet degranulation
wound healing
protein activation cascade
blood coagulation, fibrin clot formation
establishment of localization
muscle contraction
centriole replication
cytolysis
cell cycle process
positive regulation of nitric oxide biosynthetic process
heme metabolic process
positive regulation of mRNA catabolic process
activation of cysteine−type endopeptidase activity
histone phosphorylation
regulation of autophagy
cell adhesion
intrinsic apoptotic signaling pathway in response to oxidative stress
bleb assembly
hormone transport
ectoderm development
oxidation−reduction process
dorsal spinal cord development
regulation of blood pressure
Type4
Type5
Type6
Count
5
10
15
−log10(pvalue)
4.5
4.0
3.5
3.0
2.5
STr
Hipp
MD
Amy
adult locomotory behavior
chemical synaptic transmission
barbed−end actin filament capping
actin filament capping
muscle contraction
oligosaccharide catabolic process
nephron tubule development
mRNA destabilization
mitotic chromosome condensation
peptidyl−tyrosine dephosphorylation
actin filament−based process
process utilizing autophagic mechanism
cellular catabolic process
columnar/cuboidal epithelial cell development
dendritic spine maintenance
positive regulation of phosphatidylinositol 3−kinase activity
positive regulation of epithelial cell migration
substantia nigra development
microtubule−based movement
tRNA wobble uridine modification
mitotic cell cycle
positive regulation of DNA biosynthetic process
synaptic transmission, dopaminergic
spindle organization
mitotic spindle organization
chondrocyte differentiation
embryonic skeletal joint morphogenesis
epithelial cell development
bone maturation
thrombin−activated receptor signaling pathway
epithelial cell morphogenesis
cell development
cellular response to vascular endothelial growth factor stimulus
histone H2A monoubiquitination
regulation of cell adhesion mediated by integrin
cellular response to peptide hormone stimulus
cell adhesion mediated by integrin
regulation of postsynaptic membrane potential
cold−induced thermogenesis
positive regulation of synaptic plasticity
cell−cell signaling involved in cardiac conduction
mitotic centrosome separation
drug metabolic process
positive regulation of protein phosphorylation
localization of cell
tRNA modification
granulocyte differentiation
protein exit from endoplasmic reticulum
endoplasmic reticulum to cytosol transport
histone modification
carbohydrate phosphorylation
negative regulation of transcription by RNA polymerase II
regulation of cell shape
actin filament organization
ear morphogenesis
sensory organ morphogenesis
inner ear morphogenesis
Type4
Type5
Type6
Count
4
8
12
16
−log10(pvalue)
3.0
2.5
2.0

Supplementary Figure 10. Biological processes enriched by the 4-6 types genes in cortical (A) and subcortical regions (B). The color shade and dot size indicate the P value and the number of genes, respectively. The P values were calculated by two-sided Fisher's exact test (**A**, **B**). Source data are provided as a Source Data file.

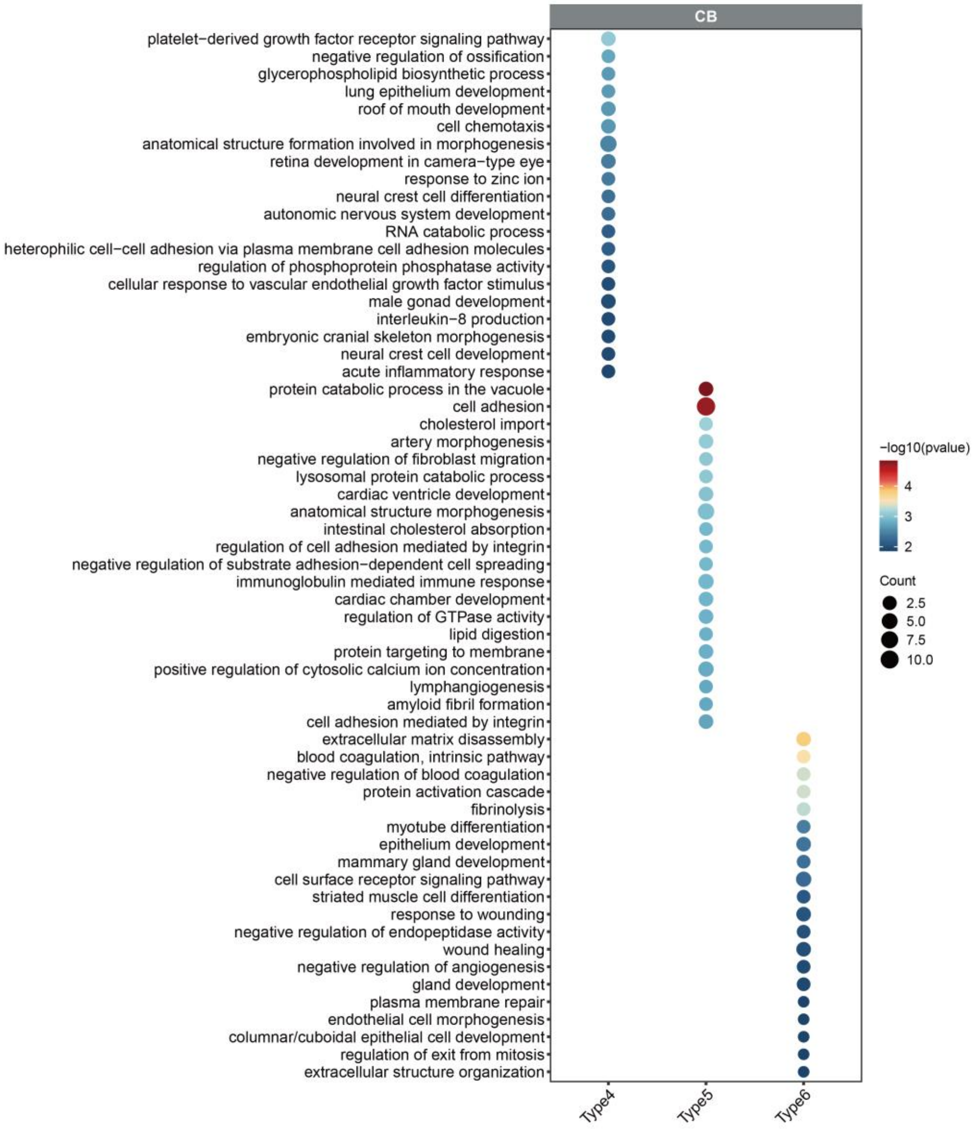

Supplementary Fig. 11. Biological processes enriched by the 4-6 types genes in cerebellum. The color shade and dot size indicate the P value and the number of genes, respectively. The P values were calculated by two-sided Fisher's exact test. Source data are provided as a Source Data file.

Supplementary Table 1. Information for the 156 samples in this study.

Animal code	*Days post-fertilization*	*Sex*	*Brain regions*	*Notes*
F50-1	50	M	PFC (FL), TL, PL, V1, CB	CS
F50-2	50	M	PFC (FL), TL, PL, V1, CB	CS
F50-3	50	M	PFC (FL), TL, PL, V1, CB	CS
F90-1	90	F	aPFC, pPFC, IC, M1, TL, PL, V1, CB, STr, Hipp, Amy	CS
F90-2	90	F	aPFC, pPFC, IC, M1, TL, PL, V1, CB, STr, Hipp, Amy	CS
F90-3	90	F	aPFC, pPFC, IC, M1, TL, PL, V1, CB, STr, Hipp, Amy	CS
F120-1	120	F	sPFG, mPFG, iPFG, OFC, IC, M1, aCG, sTG, mTG, iTG, S1, sPL, V1, CB, STr, Hipp, MD, Amy	CS
F120-2	120	M	sPFG, mPFG, iPFG, OFC, IC, M1, aCG, sTG, mTG, iTG, S1, sPL, V1, CB, STr, Hipp, MD, Amy	CS
F120-3	120	F	sPFG, mPFG, iPFG, OFC, IC, M1, aCG, sTG, mTG, iTG, S1, sPL, V1, CB, STr, Hipp, MD, Amy	CS
P3-1	150+3	F	sPFG, mPFG, iPFG, OFC, IC, M1, aCG, sTG, mTG, iTG, S1, sPL, V1, CB, STr, Hipp, MD, Amy	Born naturally
P3-2	150+3	F	sPFG, mPFG, iPFG, OFC, IC, M1, aCG, sTG, mTG, iTG, S1, sPL, V1, CB, STr, Hipp, MD, Amy	Born naturally
P3-3	150+3	M	sPFG, mPFG, iPFG, OFC, IC, M1, aCG, sTG, mTG, iTG, S1, sPL, V1, CB, STr, Hipp, MD, Amy	Born naturally

F50, F90 and 120 means 50-, 90- and 120- days post fertilization, P3 means 3 days post birth. PFC (FL): prefrontal cortex (frontal lobe), TL: temporal lobe, PL: parietal lobe, V1: primary visual cortex (occipital lobe), CB: cerebellum, aPFC: anterior prefrontal cortex, pPFC: posterior prefrontal cortex, IC: insular cortex, M1: primary motor cortex, STr: striatum, Hipp: hippocampus, sPFG: superior frontal gyrus, mPFG: middle frontal gyrus, iPFG: inferior frontal gyrus, OFC: orbital frontal cortex, aCG: anterior cingulate gyrus, sTG: superior temporal gyrus, mTG: middle temporal gyrus, iTG: inferior temporal gyrus, S1: primary somatosensory cortex, sPL: superior parietal lobule, MD: mediodorsal nucleus of the thalamus, Amy: amygdala. CS: caesarean section.
