# Supplementary material for: Spatiotemporal proteomic atlas of multiple brain regions across early fetal to neonatal stages in cynomolgus monkey
Source: Nat Commun. 2023 Jul 3;14:3917. doi: 10.1038/s41467-023-39411-7 (PMC10317979; doi:10.1038/s41467-023-39411-7)
Supplement: Supplementary file 3 — Description of Additional Supplementary Files [file 41467_2023_39411_MOESM3_ESM.pdf]

Description of Additional Supplementary Files

Supplementary Data 1. Protein expression of all samples.

Supplementary Data 2. Statistics of the identified protein in each stage and each region.

Supplementary Data 3. The percentage of subcellular localizations in different brain regions and stages.

Supplementary Data 4. The percentage of protein families in different brain regions and stages.

Supplementary Data 5. The stage-specific marker proteins (not including cerebellum) for each stage.

Supplementary Data 6. The cerebellum marker proteins over the four stages. The P values were calculated by two-sided student’s t-test test.

Supplementary Data 7. The marker proteins in cortical or subcortical region over the four stages.

Supplementary Data 8. The biological processes enriched by region-specific marker proteins over the four stages. The P values were calculated by two-sided Fisher's exact test without adjustments.

Supplementary Data 9. The biological processes enriched by the 6 types genes in cortical regions, subcortical regions and cerebellum. The P values were calculated by two-sided Fisher's exact test.

Supplementary Data 10. Raw results for the identification and quantification of all peptides and proteins.
