# Supplementary material for: Spatiotemporal proteomic atlas of multiple brain regions across early fetal to neonatal stages in cynomolgus monkey
Source: Nat Commun. 2023 Jul 3;14:3917. doi: 10.1038/s41467-023-39411-7 (PMC10317979; doi:10.1038/s41467-023-39411-7)
Supplement: Supplementary file 14 — Reporting Summary [file 41467_2023_39411_MOESM14_ESM.pdf]

nature portfolio

Corresponding author(s): Wei Si

Last updated by author(s): Mar 1, 2023

Reporting Summary

Nature Portfolio wishes to improve the reproducibility of the work that we publish. This form provides structure for consistency and transparency in reporting. For further information on Nature Portfolio policies, see our Editorial Policies and the Editorial Policy Checklist.

Statistics

For all statistical analyses, confirm that the following items are present in the figure legend, table legend, main text, or Methods section.

n/a	Confirmed	
☐	☒	The exact sample size (*n*) for each experimental group/condition, given as a discrete number and unit of measurement
☐	☒	A statement on whether measurements were taken from distinct samples or whether the same sample was measured repeatedly
☐	☒	The statistical test(s) used AND whether they are one- or two-sided<br>*Only common tests should be described solely by name; describe more complex techniques in the Methods section.*
☐	☒	A description of all covariates tested
☐	☒	A description of any assumptions or corrections, such as tests of normality and adjustment for multiple comparisons
☐	☒	A full description of the statistical parameters including central tendency (e.g. means) or other basic estimates (e.g. regression coefficient) AND variation (e.g. standard deviation) or associated estimates of uncertainty (e.g. confidence intervals)
☐	☒	For null hypothesis testing, the test statistic (e.g. *F*, *t*, *r*) with confidence intervals, effect sizes, degrees of freedom and *P* value noted<br>*Give P values as exact values whenever suitable.*
☒	☐	For Bayesian analysis, information on the choice of priors and Markov chain Monte Carlo settings
☒	☐	For hierarchical and complex designs, identification of the appropriate level for tests and full reporting of outcomes
☐	☒	Estimates of effect sizes (e.g. Cohen's *d*, Pearson's *r*), indicating how they were calculated

Our web collection on statistics for biologists contains articles on many of the points above.

Software and code

Policy information about availability of computer code

Data collection	The mass spectrometry proteomics data was collected using Q ExactiveTM Plus (ThermoFisher) coupled online to the UPLC. The Maxquant search engine (v.1.5.2.8)(https://www.maxquant.org) software was used to process proteomics data.
Data analysis	The downstream analysis and figure generation were performed by using custom R code. All analysis scripts have been deposited on GitHub (https://github.com/daishaoxing/Proteomic_BrainDev) and made publicly available via Zenodo (https://doi.org/10.5281/zenodo.7979998).

For manuscripts utilizing custom algorithms or software that are central to the research but not yet described in published literature, software must be made available to editors and reviewers. We strongly encourage code deposition in a community repository (e.g. GitHub). See the Nature Portfolio guidelines for submitting code & software for further information.

Data

Policy information about availability of data

All manuscripts must include a data availability statement. This statement should provide the following information, where applicable:

- Accession codes, unique identifiers, or web links for publicly available datasets
- A description of any restrictions on data availability
- For clinical datasets or third party data, please ensure that the statement adheres to our policy

The mass spectrometry proteomics raw data generated in this study have been deposited in the China National GeneBank DataBase (CNGBdb) under accession

code CNP0002788 and are publicly accessible at https://db.cngb.org. We provided an interactive web page (https://cpbra.cn/menu_details/cyno/cBrainDev). All processed data have also been made publicly available via Zenodo (https://doi.org/10.5281/zenodo.7979998). Source data are provided with this paper.

Human research participants

Policy information about studies involving human research participants and Sex and Gender in Research.

Reporting on sex and gender	N/A
Population characteristics	N/A
Recruitment	N/A
Ethics oversight	N/A

Note that full information on the approval of the study protocol must also be provided in the manuscript.

Field-specific reporting

Please select the one below that is the best fit for your research. If you are not sure, read the appropriate sections before making your selection.

☒ Life sciences ☐ Behavioural & social sciences ☐ Ecological, evolutionary & environmental sciences

For a reference copy of the document with all sections, see nature.com/documents/nr-reporting-summary-flat.pdf

Life sciences study design

All studies must disclose on these points even when the disclosure is negative.

Sample size	Sample sizes were not predetermined based on statistical methods, but were chosen according to previous experience and standards in the field (at least three independent biological replicates for each condition).
Data exclusions	Data were not excluded from analysis.
Replication	To ensure the robustness, we performed independent biological replicates for each experiments. There are 3 fetuses or monkeys replicates at each stage. All attempts of replication were successful and gave similar results.
Randomization	Animal subjects were randomly selected in the animal experiments.
Blinding	Blinding was not relevant to our study because in general, based on the prior experience of other groups in the field, these types of assays do not require blinding.

Reporting for specific materials, systems and methods

We require information from authors about some types of materials, experimental systems and methods used in many studies. Here, indicate whether each material, system or method listed is relevant to your study. If you are not sure if a list item applies to your research, read the appropriate section before selecting a response.

Materials & experimental systems

n/a	Involved in the study	
☐	☒	Antibodies
☒	☐	Eukaryotic cell lines
☒	☐	Palaeontology and archaeology
☐	☒	Animals and other organisms
☒	☐	Clinical data
☒	☐	Dual use research of concern

Methods

n/a	Involved in the study	
☒	☐	ChIP-seq
☒	☐	Flow cytometry
☒	☐	MRI-based neuroimaging

Antibodies

Antibodies used	Primary antibodies: Sox2 rabbit (Millipore, AB5603, 1: 500); Neurocan (NCAN) mouse (Abcam, ab31979, 1: 500).<br>Secondary antibodies: Goat anti-rabbit IgG (H+L) Cross-Adsorbed Secondary Antibody (Invitrogen, A11012, 1:500) with Alexa Fluor

	594; Goat anti-mouse IgG (H+L) Highly Cross-Adsorbed Secondary Antibody (Invitrogen, A11029, 1:500) with Alexa Fluor 488. DAPI: Mounting agent with DAPI - Aqueous Fluoroshield (Abcam, ab104139, 1:500).
Validation	All antibodies used in this work were purchased from companies, and validated by the manufacturers and by extensive use in published work.

Animals and other research organisms

Policy information about studies involving animals; ARRIVE guidelines recommended for reporting animal research, and Sex and Gender in Research

Laboratory animals	Female and male fetuses of cynomolgus monkeys (Macaca fascicularis) of 50, 90 and 120 days post-fertilization and female and male newborn cynomolgus monkeys of 3 days were used.
Wild animals	The study did not involve wild animals.
Reporting on sex	This study contains 3 male monkey fetuses of 50 days post-fertilization (F50), 3 female F90 monkey fetuses, 2 female and 1 male F120 monkey fetuses and 2 female and 1 male new born monkeys.
Field-collected samples	The study did not involve samples collected from the field.
Ethics oversight	The parents of 9 fetuses and 3 postnatal monkeys were individually raised in an animal room with humidity at 40-70%, temperature at 18-26 °C and 12/12 light–dark cycle. The experimental plan was approved by the Institutional Animal Care and Use Committee of Kunming University of Science and Technology in advance (approval number: LPBR201801020). All procedures involving animals were performed in accordance with the Guide for the Care and Use of Laboratory Animals (the 8th edition, NIH).

Note that full information on the approval of the study protocol must also be provided in the manuscript.
